# BRCAness in prostate cancer

**DOI:** 10.18632/oncotarget.26818

**Published:** 2019-03-29

**Authors:** Natalia Bednarz-Knoll, Elke Eltze, Axel Semjonow, Burkhard Brandt

**Affiliations:** Natalia Bednarz-Knoll: Laboratory of Cell Biology, Department of Medical Biotechnology, Medical University of Gdańsk, Gdańsk, Poland

**Keywords:** prostate cancer, tumor progression, tumor marker, BRCA1, BRCAness

The aim of early detection of prostate cancer (PCa) is to improve quality of life and decrease PCa-associated mortality. The use of prostate specific antigen (PSA) in a population-based screening study resulted in an increased incidence of PCa, a shift toward earlier disease stages and a reduction of metastatic disease and deaths from PCa. However, this benefit is accompanied by considerable rates of overdiagnosis which also involves potential overtreatment [1]. In consequence, biomarkers are urgently needed that indicate the metastatic potential of a tumor at the time of primary diagnosis. Our data based on genotyping of local tumors, lymph nodes and corresponding circulating tumor cells (CTCs) made it reasonable to assume that cells from distinct loci of heterogenous PCa spread to other organs long before the overt manifestation of metastases [3–5]. Even small foci of cells bearing predominantly BRCA1 and PTEN gene losses can initiate PCa cell regional and distant dissemination, indicating those patients at high risk of metastasis [2, 3]. Additionally, androgen receptor (AR) expression and AR-dependent proteins are upregulated in PTEN gene deficient tumors [4]. Finally, our data are in accordance to the recent whole genome sequencing data for untreated PCa metastasis indicating that a large majority of driver gene mutations are common to all metastases [6].

BRCA1 dysfunction causes impairment of DNA repair mechanisms called homologous recombination (HR). This holds true also in tumors that do not harbour hereditary BRCA1 gene mutation. Moreover, this new concept called “BRCAness” [7] was extended to defects in other similar genes [3, 4]. Simultaneously, HR in cancers with BRCA mutations became a therapy target with poly(ADP-ribose) polymerases (PARP) inhibitors and are already studied in clinical trials of PCa [8]. There is an additional upcoming need for those markers by extending PARP inhibitor therapy to BRCAness tumors driven by genes, other than BRCA genes. Our studies show new candidate genes (PTEN, ALDH1, EGFR, PAPSS2), that could refine the prediction of response to such therapy when determined in biopsies, CTCs and even cell-free DNA in plasma.

In male, BRCA1/2 germline mutations are reported to increase the risk of PCa and correlate to its more aggressive course [9]. Somatic BRCA1 gene losses are found in approx. 1/10^th^ of PCa [2, 5]. Interestingly, our recent study suggested that also BRCA1 gene gains might account for BRCAness, as a part of tumors characterized by this type of genetic alteration revealed lack of functional BRCA1 protein [5].

There is some evidence, that the hierarchical model of breast cancer development might be applicable in PCa. Thereby, accumulation of so called cancer stem or progenitor cells induced by bi- or monoallelic loss of BRCA1 gene [10] leads to therapy resistance and/or metastatic spread. We showed that loss of BRCA1 is associated with advanced PCa conceivably indicating cancer stem cell phenotype, determined by expression of ALDH1 and EGFR [5]. In addition, BRCAness may also correlate with another process frequently linked to cancer stem cell phenotype, i.e. epithelial-mesenchymal transition (EMT). EMT-related signatures such as vimentin or EGFR were coexpressed in PCa with BRCA1 gene losses [2, 5]. Thus, a similar role for BRCA1 in PCa as already described for breast cancer could be assumed for the induction of phenotype plasticity and migration [11].

In conclusion, BRCAness in prostate cancer has the potential to indicate PCa patients with the highest risk of metastasis [4]. Nevertheless, further studies are needed to evaluate candidate genes for early PCa detection and therapy resistance as well as to establish robust assays for detection.
